# Biological Pathways Associated With the Development of Pulmonary Toxicities in Mesothelioma Patients Treated With Radical Hemithoracic Radiation Therapy: A Preliminary Study

**DOI:** 10.3389/fonc.2021.784081

**Published:** 2021-12-22

**Authors:** Sergio Crovella, Alberto Revelant, Elena Muraro, Ronald Rodrigues Moura, Lucas Brandão, Marco Trovò, Agostino Steffan, Paola Zacchi, Giuliano Zabucchi, Emilio Minatel, Violetta Borelli

**Affiliations:** ^1^ Department of Biological and Environmental Sciences, College of Arts and Sciences, University of Qatar, Doha, Qatar; ^2^ Department of Radiation Oncology, Centro di Riferimento Oncologico di Aviano (CRO) Istituti di Ricovero e Cura a Carattere Scientifico (IRCCS), Aviano, Italy; ^3^ Immunopathology and Biomarker Unit, Department of Translational Research, Centro di Riferimento Oncologico di Aviano (CRO) Istituti di Ricovero e Cura a Carattere Scientifico, Aviano, Italy; ^4^ Department of Advanced Diagnostics, Institute for Maternal and Child Health – Istituti di Ricovero e Cura a Carattere Scientifico (IRCCS) “Burlo Garofolo”, Trieste, Italy; ^5^ Radiation Oncology Department, Udine Academic Hospital, Udine, Italy; ^6^ Department of Life Sciences, University of Trieste, Trieste, Italy

**Keywords:** mesothelioma, radical hemithoracic radiotherapy, pulmonary toxicities, whole exome, thromboembolism

## Abstract

Radical hemithoracic radiotherapy (RHR), after lung-sparing surgery, has recently become a concrete therapeutic option for malignant pleural mesothelioma (MPM), an asbestos-related, highly aggressive tumor with increasing incidence and poor prognosis. Although the toxicity associated to this treatment has been reduced, it is still not negligible and must be considered when treating patients. Genetic factors appear to play a role determining radiotherapy toxicity. The aim of this study is the identification of biological pathways, retrieved through whole exome sequencing (WES), possibly associated to the development of lung adverse effects in MPM patients treated with RHR. The study included individuals with MPM, treated with lung-sparing surgery and chemotherapy, followed by RHR with curative intent, and followed up prospectively for development of pulmonary toxicity. Due to the strong impact of grade 3 pulmonary toxicities on the quality of life, compared with less serious adverse events, for genetic analyses, patients were divided into a none or tolerable pulmonary toxicity (NoSTox) group (grade ≤2) and a severe pulmonary toxicity (STox) group (grade = 3). Variant enrichment analysis allowed us to identify different pathway signatures characterizing NoSTox and Stox patients, allowing to formulate hypotheses on the protection from side effects derived from radiotherapy as well as factors predisposing to a worst response to the treatment. Our findings, being aware of the small number of patients analyzed, could be considered a starting point for the definition of a panel of pathways, possibly helpful in the management of MPM patients.

## 1 Introduction

Malignant pleural mesothelioma (MPM) is an asbestos-related, highly aggressive tumor derived from cells of the pleura, with increasing incidence (expected to peak in the period 2020–2024) (1) and poor prognosis. Such dismal outcome also derives from the complex management of the disease, given that the scientific community still debates about the best protocol to follow based on the three therapeutic options that are currently available: surgery, radiotherapy, and chemotherapy (2–4).

Until recently, it was thought that the efficacy of routine administration of radiotherapy in patients with MPM was not supported by evidence (5). In recent years, modern radiation techniques have been applied postsurgery, allowing for more effective sparing of normal tissue, thus enabling higher radiation doses at the tumor site. Reasearchers from the National Cancer Institute of Aviano have published a series of prospective studies (6–8) proving that radical intensity-modulated radiation therapy, after lung-sparing surgery, leads to excellent loco-regional control and survival in MPM patients. A median overall survival (OS) of 25.6 months and a 2-year OS rate of 58% are among the best results observed in recent studies (9–11), supporting the idea that this approach represents a concrete therapeutic option for MPM.

Although the toxicity associated to these treatments has been drastically reduced, it is still not negligible and must be taken into consideration when treating patients (12). Pulmonary toxicities are very common among mesothelioma patients undergoing radiotherapy on the entire hemithorax even if the treatment is well tolerated. Albeit infrequent, worse adverse effects, like grade 2 or 3 fibrosis and pulmonary embolism, can be seen, and the management of these toxicities is still challenging.

Improved ways of predicting, prior to treatment, the risk of development of adverse lung effects after radiotherapy may result in more promising personalized treatments and in a reduced incidence and severity of late effects. There is increasing recognition that the cause of normal tissue toxicity is multifactorial and includes genetic factors in addition to dosimetric parameters, patient age, smoking history, concurrent treatments, and comorbidities (13–15).

In the last decade, more than one hundred papers have been published addressing possible associations between genetic germline variants and risk of normal tissue toxicity after radiotherapy. With few exceptions, however, these relied on relatively few studies that used a candidate gene approach [single nucleotide variant (SNV) analysis] (16), and the association results have not been replicated (17, 18).

Recently, genome-wide association analyses (GWAS), including meta-analysis, performed by the Radiogenomics Consortium (RgC; epi.grants.cancer.gov/radiogenomics/) have identified several novel SNVs within genes not previously linked to radiotherapy toxicity, in patients affected by different types of cancers, such as breast, prostate and lung (19, 20). Nevertheless, genetic susceptibility to radiotoxicity in nonsyndromic individuals remains to be unravelled. Aimed at contributing to the identification of a possible role for genetic variations in the biological pathways involved in the response to radiotherapy in MPM patients, we performed whole exome sequencing (WES), coupled with a novel bioinformatic approach, focusing not only on SNVs but especially on potential biological pathways associated to pulmonary toxicity after radiotherapy that can help to better elucidate the observed phenotypes.

## 2 Methods

### 2.1 Study Participants

The study included individuals with MPM, treated with nonradical surgical treatment, such as partial pleurectomy/decortication (P/D) or biopsy only, and platinum-based plus pemetrexed chemotherapy for MPM, followed by high-dose radiation therapy with curative intent, and followed up prospectively for development of (pulmonary) toxicity. Other inclusion criteria were age ≥18 years, proven gross residual disease after surgery, stages I–IVA (according to TNM stage 7th edition), Eastern Cooperative Oncology Group (ECOG) performance status scores 0–2, pulmonary function of at least 50% of predicted, technical feasibility for delivery of radical hemithoracic radiotherapy (RHR), satisfactory bone marrow function (white blood cells ≥2,000/μl, platelets ≥100,000/μl, haemoglobin >10 g/dl). Exclusion criteria were pathologic contralateral mediastinal nodes (N3), metastatic MPM (stage IVB), or intra-scissural disease. Tumour histology was classified as epithelioid and non-epithelioid (sarcomatoid and biphasic). All patients were staged by lung and abdomen contrast-enhanced computed tomography (CT) scans and a 18F-fluorodeoxyglucose (18FDG)-positron emission tomography (PET)/CT.

Between August 2014 and May 2018, 49 patients who received RHR were included in this study. Patients were treated with helical IMRT, delivering the dose with Accuray Tomotherapy System. The radiation therapy technique has previously been described in detail (7, 8, 11). Patients received 50 Gy in 25 fractions (except for one case, later described), plus an eventual boost to 60 Gy on PET-positive areas.

OS was defined as the time (years) intercourse since randomization to death from any causes, or the last follow-up (until December 2020, 1st), and estimated with the Kaplan-Meier method, *p*-value was calculated with Log-rank test.

This prospective study was conducted according to the ethical principles of the Declaration of Helsinki and approved by the local Ethical Committee (*Comitato Etico Indipendente del CRO di Aviano*, CRO-2013-38) and written informed consent was obtained from all the patients. The CRO-biobanking service managed and stored all biological samples before use for the present project (authorization for analyses obtained through protocol number 6825/D).

### 2.2 Assessment of Radiotherapy Toxicity

Toxicity was assessed using the Common Terminology Criteria for Adverse Events version 3.0, and divided into early toxicity (during treatment), acute toxicity (1–6 months from the end of RHR), and late toxicity (>6 months from the end of RHR).

Early toxicity was assessed weekly during radiation. Following completion of radiation treatment, patients were reviewed at 1, 3, 6 (acute toxicity), 8, and 12 months in the first year (late toxicity) and every 4 months from the start of second year or before for clinical need. All respiratory toxicities outcomes were analyzed: cough, dyspnea, fibrosis, pulmonitis, and pulmonary thromboembolic events.

### 2.3 Genotyping, Quality Control, and Imputation/Statistical Analysis

#### 2.3.1 DNA Extraction

Germline DNA from whole blood (blood samples collected in anticoagulant-citrate-dextrose before the start of radiation treatment) was extracted from patients using the DNeasy Blood & Tissue Kit (Qiagen, Milan, Italy) following the manufacturer’s protocol. DNA quality and quantity were evaluated by agarose gel (2%) electrophoresis and using the Invitrogen Qubit assay (Thermo Fisher Scientific, Milan, Italy). All samples (*N* = 49) extracted successfully passed the quality control, based on Macrogen (https://dna.macrogen-europe.com/eng/) requirements for exome sequencing.

#### 2.3.2 Exome Sequencing

Exome sequencing was performed in outsourcing using the service provided by Macrogen Europe (Amsterdam, The Netherlands). Briefly, the exome sequencing analysis, aiming at 150× coverage, used the Illumina^®^ SureSelect Human V7 Kit Library preparation and sequencing reaction, in an Illumina^®^ HiSeq 2500 System, generating pair-end reads of 125 base pairs.

Illumina universal adapters were removed using Trim Galore 0.6.1 (http://www.bioinformatics.babraham.ac.uk/projects/trim_galore/). Reads with length below 15 base pairs and with low Phred score (*Q <*20) were also removed using the same software. Quality control before and after these procedures were evaluated by fastQC (https://www.bioinformatics.babraham.ac.uk/projects/fastqc/).

After QC, the FASTQ file with the raw reads was aligned using Burrows-Wheeler Aligner Software Package (21), specifically the bwa-mem tool, against the Reference Human Genome version 38 (GRCh.38). We then used Picard tools (https://broadinstitute.github.io/picard/) to mark duplicates, and GATK V. 4.1.2.0 (https://software.broadinstitute.org/gatk/) for base recalibration. Strelka2 was used for variant calling, when the software was employed in the exome mode of analysis (22). Finally, we used GATK again for the exclusion of low-quality variants.

Variant annotation was performed using the ANNOVAR (23) software with databases relative to the GRCh.38 reference genome (refGene, cytoBand, wgRna, 1000g_2015_aug_all, gnomad30_genome, dbscsnv11, dbnsfp35a, clinvar_20200316, and avsnp151). R Software (24) was employed to manipulate ANNOVAR results for a descriptive and inferential analysis.

We divided the analysis into two main parts: individual, which consists in summarizing the descriptive data for each sequenced sample; and group data, which aims at drawing comparisons between patients with severe toxity versus no severe toxity. Principal component analysis (PCA) of variant distribution was performed using stats R packages in a noncentered approach and scaled to have unit variance before the analysis (25).

In addition, for both individual and group categories, we performed a “variant enrichment analysis” (VEA). VEA works similarly to gene enrichment analysis for expression data: we investigated if there were statistical differences between the numbers of variants in a pathway compared with a reference dataset. We used the R package “ReactomePA” to obtain pathway information on each of the genes containing at least one variant in the individual dataset (26). Then, we obtained variant data from GnomAD Exome 3.0 (27) to use as a reference dataset. It is important to mention that, since the occurrence of some variants may be related to specific populations, we considered for this dataset only the Non-Finnish European (nfe) variant information.

Once we had the number of variants per pathway in the individual dataset and in the reference dataset, we used Fisher’s exact test with false discovery rate (FDR) to identify statistical differences in the number of variants. Adjusted *p*-values <0.05 were considered significant in this analysis. For group data, we used Venn diagrams to summarize “enriched” pathways exclusive for each group.

## 3 Results

### 3.1 Patients

Patients’ characteristics are summarized in **Table 1**. A total of 48 patients completed the treatment as prescribed. One patient, requiring a treatment break due to anemia and trombocytopenia, globally received 32 Gy in 16 fractions. One patient developed infective pneumonia independent of RT, and discontinued the treatment, completing the prescribed course at a later moment. Forty-five patients received the simultaneous boost.

**Table 1:** Patients and tumor characteristics.

Characteristic	Value	%
Gender		
Male	40	81.6
Female	9	18.4
Age (years)		
Median	69	NA
Range	33–81	NA
Laterality		
Right	26	53.0
Left	21	42.9
nd	2	4.1
Histological subtype (*n*)		
Epithelioid	46	93.9
Nonepithelioid	3	6.1
Pathological stage		
Stages I–II	20	40.8
Stages III–IV	29	59.2

NA, not applicable.

Reported adverse effects are summarized in **Table 2**, divided in events observed during treatment (early toxicity), between 1 and 6 months after the end of RT (acute toxicity), and after 6 months of follow-up (late toxicity), and classified as nonpulmonary or pulmonary toxicities.

**Table 2:** Nonpulmonary and pulmonary events observed as early, acute, and late toxicities.

Nonpulmonary toxicity	Early toxicity [*n* (%)]	Acute toxicity [*n* (%)]	Late toxicity [*n* (%)]
Anemia			
• Grades 1–2	0 (0)	0 (0)	1 (2)
• Grade 3	1 (2)	0 (0)	0 (0)
Chest wall pain			
• Grades 1–2	12 (24)	12 (24)	5 (10)
• Grade 3	1 (2)	0 (0)	1 (2)
Dermatitis			
• Grades 1–2	11 (22)	2 (4)	0(0)
• Grade ≥3	1 (2)	0 (0)	0(0)
Fatigue			
• Grades 1–2	19 (39)	10 (20)	4 (8)
• Grade 3	1 (2)	1 (2)	1 (2)
Fracture	0 (0)	0(0)	2 (4)
GGT			
• Grades 1–2	9 (18)	11 (22)	5 (10)
• Grade 3	0 (0)	1 (2)	3 (6)
Nausea/Vomiting			
• Grades 1–2	23 (47)	1 (2)	1 (2)
• Grade 3	0 (0)	0 (0)	0 (0)
Oesophagitis			
• Grades 1–2	34 (69)	6 (12)	1 (2)
• Grade 3	2 (4)	0 (0)	0 (0)
Pericardial effusion			
• Grades 1–2	0 (0)	0(0)	0 (0)
• Grade 3		1(2)	2 (4)
**Pulmonary toxicity**			
Cough			
• Grades 1–2	5 (10)	16 (33)	14 (29)
• Grade 3	0 (0)	0 (0)	0 (0)
Dyspnea			
• Grades 1–2	9 (18)	17 (35)	7 (14)
• Grade 3	0 (0)	1 (2)	4 (8)
Fibrosis			
• Grades 1–2	N.A.	0(0)	1 (2)
• Grade 3		1(2)	1 (2)
Pneumonitis			
• Grades 1–2	1 (2)	1 (2)	1 (2)
• Grade 3	0 (0)	2 (4)	2 (4)
Thromboembolic event	0 (0)	2 (4)	1 (2)

GTT, gamma-glutamyltransferase; N.A., Not Applicable.

Globally, for acute and late toxicities, the number of events in the nonpulmonary toxicity group tended to diminish, except for the rise in liver gamma-glutamyltransferase (GGT), commonly observed in patients treated on the right hemithorax (not shown). Fractures and pericardial effusions are typically late events and were observed in a small number of patients, 2 and 3 cases, respectively. Conversely, pulmonary toxicity events tended to develop after treatment and to last in time. One-third of the patients developed low-grade cough by 6 months after RT, or at a later follow-up. Dyspnea was reported in 37% of patients as acute toxicity, mainly low grade, but became a severe toxicity in 8% of patients, as a late event. Fibrosis developed in 3 patients (2 severe), and pneumonitis in 7 cases, 4 of which were grade 3. Finally, 3 thromboembolic events were reported after treatment.

Due to the impact of grade 3 pulmonary toxicities on the quality of life, for further genetic analyses patients were divided into two groups: no severeTox (NoSTox), none or tolerable toxicity (grade 0 to 2) (grade ≤2) (*N* = 40), and severeTox (STox) (grade = 3) (*N* = 9) based on the development of at least one among the following: cough, dyspnea, fibrosis, pneumonitis, and/or thromboembolic event. STox patients’ characteristics and reported adverse effects are summarized in **Table 3**. No grade 4 or 5 toxicities were recorded during treatment.

**Table 3:** STox: patient, tumor and pulmonary toxicities characteristics.

Patient ID#	Gender	Age	Laterality	Histological subtype	Pathological stage		Early toxicity	Acute toxicity	Late toxicity
					T stage	N stage			
2	M	71	Right	Epithelioid	4	0	None	Dyspnea G2, pneumonitis G3, cough G2, thromboembolic event G3	Dyspnea G3
12	M	63	Left	Epithelioid	2	0	Pneumonitis G1	Fibrosis G3	None
17	M	72	Left	Epithelioid	2	0	None	None	Thromboembolic event G3
20	F	76	Right	Epithelioid	3	0	None	None	Pneumonitis G3, cough G2
53	M	65	Right	Epithelioid	2	0	None	None	Cough G2, dyspnea G2, pneumonitis G3
58	F	65	Left	Epithelioid	3	2	None	Dyspnea G3	Cough G2, pneumonitis G3, dyspnea G3,
61	F	72	Right	Epithelioid	2	0	None	Dyspnea G2	Cough G2, dyspnea G3
70	M	78	Left	Epithelioid	3	0	Dyspnea G2	Pneumonitis G3, thromboembolic event G3	None
94	M	75	Left	Epithelioid	3	0	Dyspnea G1	Dyspnea G2	Pneumonitis G2, fibrosis G3

Among the 49 patients included, median OS was 25 months, and 2-year OS rate was 51.4%. No significative survival differences were observed between the NoSTox group and the STox group (data not shown).

### 3.2 Genetic Analysis by Variant Enrichment Analysis

The global genetic analysis results and all exome detailed reports are shown in **Supplementary Datas 1, 2**. **Supplementary Data 1** contain the descriptive information about exomic analysis of the 49 patients with MPM; the distribution of variants (**Supplementary Table S1A**), distribution (%) of variant location (**Supplementary Figure S1A**), function (**Supplementary Figure S1B**), and distribution of genes carrying variants (**Supplementary Table S1B**) are reported in the exome report of both groups (STox and NoSTox). **Supplementary Data 2** contain a comparative description about genes and common variants of 49 patients with MPM; NoSTox and STox common variants were filtered to retrieve only the common FunctVar (**Supplementary Table S2A**) and ImpactVar (**Supplementary Table S2B**) variants exclusive of the STox group; similarly, NoSTox and STox common variants were filtered to retrieve the common FunctVar (**Supplementary Table S2C**) and ImpactVar (**Supplementary Table S2D**) variants present just in the NoSTox group. Noteworthy, none of the genes reported by Hylebos and colleagues (28), to exhibit molecular alterations in MPM, were related with the development of pulmonary toxicity in the subjects involved in this study (data not shown).

Based on the results obtained considering variants present just in the STox patients, we retrieved the pathways possibly disrupted by the identified genetic variants, without, however, detecting a specific pathway signature associated with the clinical phenotypes observed (data not shown).

Furthermore, with the aim of applying a wider approach to enrich the pathway analysis, we performed a variant enrichment analysis based on functional and impact variants; 47 pathways showed a significantly increased number of functional variants exclusively in the NoSTox group (**Table 4**). For the STox group, we observed four exclusive pathways (**Table 5**). Regarding the impact variants, we observed 19 and 3 exclusive pathways for the NoSTox and STox groups, respectively (**Tables 6**, **7**).

**Table 4:** List of the reactome pathways retrived from variant enrichement analysis (VEA) for functional variant in the NoSTox group.

DB_ID	Pathway name	Patient ID#	Number of patients
R-HSA-73776	RNA polymerase II promoter escape	x7,x22,x55	3
R-HSA-73779	RNA polymerase II transcription preinitiation and promoter opening	x7,x22,x55	3
R-HSA-75953	RNA polymerase II transcription initiation	x7,x22,x55	3
R-HSA-76042	RNA polymerase II transcription initiation and promoter clearance	x7,x22,x55	3
R-HSA-72200	mRNA editing: C to U conversion	x9,x49,x29	3
R-HSA-75072	mRNA editing	x9,x49,x29	3
R-HSA-75094	Formation of the editosome	x9,x49,x29	3
R-HSA-73780	RNA polymerase III chain elongation	x10	1
R-HSA-73980	RNA polymerase III transcription termination	x10	1
R-HSA-74158	RNA polymerase III transcription	x10,x32	2
R-HSA-749476	RNA polymerase III abortive and retractive initiation	x10,x32	2
R-HSA-76046	RNA polymerase III transcription initiation	x10,x32	2
R-HSA-76061	RNA polymerase III transcription initiation from type 1 promoter	x10,x32	2
R-HSA-76066	RNA polymerase III transcription initiation from type 2 promoter	x10,x32	2
R-HSA-76071	RNA polymerase III transcription initiation from type 3 promoter	x10,x32	2
R-HSA-8850843	Phosphate bond hydrolysis by NTPDase proteins	x14	1
R-HSA-75105	Fatty acyl-CoA biosynthesis	x15	1
R-HSA-73728	RNA polymerase I promoter opening	x21,x39,x43	3
R-HSA-74749	Signal attenuation	x21,x39,x43	3
R-HSA-74751	Insulin receptor signaling cascade	x21,x39,x43,x16	4
R-HSA-879415	Advanced glycosylation endproduct receptor signaling	x21,x39,x43	3
R-HSA-881907	Gastrin-CREB signaling pathway *via* PKC and MAPK	x21,x39,x43	3
R-HSA-8853659	RET signaling	x21,x50	2
R-HSA-77075	RNA Pol II CTD phosphorylation and interaction with CE	x22,x55	2
R-HSA-879518	Transport of organic anions	x43,x48	2
R-HSA-8852405	Signaling by MST1	x43,x48,x79	3
R-HSA-73942	DNA damage reversal	x62,x82,x85	3
R-HSA-73943	Reversal of alkylation damage by DNA dioxygenases	x62,x82,x85	3
R-HSA-75109	Triglyceride biosynthesis	x62,x77,x96	3
R-HSA-8848021	Signaling by PTK6	x62,x74	2
R-HSA-8849474	PTK6 activates STAT3	x62	1
R-HSA-5357956	TNFR1-induced NFkappaB signaling pathway	x74	1
R-HSA-5358346	Hedgehog ligand biogenesis	x74	1
R-HSA-5362768	Hh mutants that do not undergo autocatalytic processing are degraded by ERAD	x74	1
R-HSA-73893	DNA damage bypass	x74,x37	2
R-HSA-75815	Ubiquitin-dependent degradation of Cyclin D	x74	1
R-HSA-8849469	PTK6 regulates RTKs and their effectors AKT1 and DOK1	x74	1
R-HSA-8852276	The role of GTSE1 in G2/M progression after G2 checkpoint	x74,x29	2
R-HSA-8854050	FBXL7 downregulates AURKA during mitotic entry and in early mitosis	x74	1
R-HSA-75035	Chk1/Chk2(Cds1)-mediated inactivation of cyclin B:Cdk1 complex	x84	1
R-HSA-73614	Pyrimidine salvage	x87	1
R-HSA-804914	Transport of fatty acids	x87	1
R-HSA-73843	5-Phosphoribose 1-diphosphate biosynthesis	x92,x37,x69	3
R-HSA-844615	The AIM2 inflammasome	x16	1
R-HSA-8862803	Deregulated CDK5 triggers multiple neurodegenerative pathways in Alzheimer’s disease models	x56	1
R-HSA-72764	Eukaryotic translation termination	x88	1
R-HSA-5358493	Synthesis of diphthamide-EEF2	x96	1

**Table 5:** List of the reactome pathways retrived from variant enrichement analysis (VEA) for functional variant in the STox group.

DB ID	Pathway name	Patient ID#	Number of patients
R-HSA-75205	Dissolution of fibrin dissolution of fibrin clot	x2	1
R-HSA-72649	Translation initiation complex formation	x17,x53	2
R-HSA-72695	Formation of the ternary complex, and subsequently, the 43S complex	x53	1
R-HSA-72702	Ribosomal scanning and start codon recognition	x53	1

**Table 6:** List of the reactome pathways retrived from variant enrichement analysis (VEA) for impact variant in the NoSTox group.

DB ID	Pathway name	Patient ID#	Number of patients
R-HSA-75035	Chk1/Chk2(Cds1)-mediated inactivation of cyclin B:Cdk1 complex	x14,x15,x19,x31,x43,x48,x84, x85,x87,x37,x50	11
R-HSA-8849469	PTK6 regulates RTKs and their effectors AKT1 and DOK1	x14,x62,x55,x88	4
R-HSA-202403	TCR signaling	x31	1
R-HSA-202424	Downstream TCR signaling	x31,x16	2
R-HSA-202427	Phosphorylation of CD3 and TCR zeta chains	x31,x16	2
R-HSA-202430	Translocation of ZAP-70 to immunological synapse	x31,x85,x16	3
R-HSA-202433	Generation of second messenger molecules	x31,x16	2
R-HSA-388841	Costimulation by the CD28 family	x31	1
R-HSA-389948	PD-1 signaling	x31,x16	2
R-HSA-8853336	Signaling by plasma membrane FGFR1 fusions	x39,x52,x56	3
R-HSA-8849474	PTK6 activates STAT3	x62,x55	2
R-HSA-198933	Immunoregulatory interactions between a lymphoid and a nonlymphoid cell	x74	1
R-HSA-6805567	Keratinization	x84	1
R-HSA-844615	The AIM2 inflammasome	x84	1
R-HSA-75896	Plasmalogen biosynthesis	x50,x66,x88	3
R-HSA-8849468	PTK6 regulates proteins involved in RNA processing	x55,x59,x88	3
R-HSA-977347	Serine biosynthesis	x71	1
R-HSA-381753	Olfactory signaling pathway	x96	1
R-HSA-8853333	Signaling by FGFR2 fusions	x96	1

**Table 7:** List of the reactome pathways retrived from variant enrichement analysis (VEA) for impact variant in the STox group.

DB ID	Pathway name	Patient ID#	Number of patients
R-HSA-75064	mRNA editing: A to I conversion	x94	1
R-HSA-75102	C6 deamination of adenosine	x94	1
R-HSA-77042	Formation of editosomes by ADAR proteins	x94	1

Tracing back the variants possibly involved in those pathways, we noticed 243 functional variants (along 64 genes), for the NoSTox group, and 9 variants for the STox (in 5 genes) (**Supplementary Data 3**: **Table S3A, B**). When considering the pathways with impact variants, we identified 2,908 variants (in 645 genes) present in the pathways exclusive of the NoSTox group, while only one variant (one gene) was observed in the pathways of the STox group (**Supplementary Data 3**: **Tables S3C, D**).

Despite the genetic differences and the number of diverse variants found between NoSTox and STox, principal component analysis (PCA) showed a homogeneous distribution of variants in both groups. Thus, no specific genetic signature was observed in NoSTox and STox (**Supplementary Figure S3**). However, in all analyses, patient 53 showed relevant significant genetic distance compared with all other patients regardless of whether they were NoSTox or STox, suggesting the presence of a specific genetic signature, but not influencing the response to radiotherapy.

#### 3.2.1 NoSTox Variant Enrichment Analysis

The variant enrichment analysis allowed us to identify pathways in the NoSTox group mainly involved in common and nonspecific biological processes such as RNA processing, cell signaling, cell cycle, and lipid synthesis. Within the identified pathways, four of them, related to inflammatory and fibrotic processes, possibly involved in the development of lung toxicity, were also found as *in silico* disrupted based on functional or impact variants not presented (or rare) in GNOMAD 2.2.1 Non-Finnish Population database (marked in grey in **Tables 4**, **6** and listed in **Table 8**).

1) The first one is the TNFR1-induced NFkappaB signaling pathway (R-HSA-5357956). One functional variant in this pathway was found in patient #74: a frameshift insertion in the ubiquitin C (UBC) gene (ENTREZID: 7316; exon 2: c.2051dupG or p.V685Cfs*7; without rs ID; located on chr12 at 124911720 position) in heterozygosis.
2) The second one is the AIM2 inflammasome pathway (R-HSA-844615). A single functional variant related to this pathway was observed in patient #16: a frameshift deletion in the Absent in melanoma 2 (AIM2) gene (ENTREZID: 9447; exon 5: c.712delA or p.T238Hfs*14; without rs ID; located on chr1 at 159062697 position) (**Table 8**). Notably, this pathway was also found among the list of impact reactome pathways in a different patient who did not develop severe pulmonary toxicities (#84, **Table 6**). In this case, patient #84 bore a variant on exon2 (c.C278G) of PYD and CARD Domain Containing (PYCARD) (ENTREZ ID: 29108; variant without rs ID located on chr 16 at position 31202200).
3) The third pathway identified as impaired in the NoSTox group, is the insulin receptor signaling cascade pathway (R-HSA-74751). Three frameshift and one stopgain functional variants were found in patients #21, #39, #43, and #16, respectively (**Table 8**). All frameshift variants were in the mitogen-activated protein kinase 3 (MAPK3) gene (ENTREZ ID: 5595). Patients #39 and #43 carried the variant on exon1 c.37_38insC (without rs ID, located on chr16 at position 30123172). Patient #21 carried one variant on exon 1 c.38_39insC (without rs ID; located on chr16 at position 30123171). The stopgain variant of Patient #16 was located on exon1 of the Phosphodiesterase 3B (PDE3B) gene (ENTREZ ID: 5140; exon 1 c.C447A; whitout rs ID; located chr11 at 14644522 position).
4) The last pathway predicted to be impaired in the NoSTox group is related to plasmalogen biosynthesis (R-HSA-75896), in patients #50, #66, and #88 (**Table 8**). The gene glyceronephosphate O-Acyltransferase (GNPAT) (ENTREZ ID: 8443) showed impact variations in patients #50 and #66, rs11122266 and rs767514222, respectively, both in exon 9. Patient #88 carried the rs764286061 SNP in the alkylglycerone phosphate synthase (AGPS) gene (ENTREZ ID: 8540).

**Table 8:** List of the selected reactome pathways retrived from variant enrichement analysis (VEA) for functional and impact variant in the NoSTox and STox groups: patients, genes and characteristics of variants involved.

DB_ID	Pathway name	Patient ID#	Gene	ENTREZ ID	Exon/Change	rs ID	QUAL/AD	Chr	Position	Type	Impact score/CADD
**NoSTox group**												
**Functional variants**												
R-HSA-74751	Insulin receptor signaling cascade	21	MAPK3	5595	1/c.38_39insC	NA	44/104	16	30123171	Frameshift	NA
		39	MAPK3	5595	1/c.37_38insC	NA	206/175	16	30123172	Frameshift	NA
		43	MAPK3	5595	1/c.37_38insC	NA	58/110	16	30123172	Frameshift	NA
		16	Phosphodiesterase 3B (PDE3B)	5140	1/c.C447A	NA	307/116	11	14644522	Stopgain	1/35
R-HSA-5357956	TNFR1-induced NFkB signaling pathway	74	Ubiquitin C (UBC)	7316	2/c.2051dupG	NA	1343/198	12	124911720	Frameshift insertion	NA
R-HSA-844615	The AIM2 inflammasome	16	Absent in melanoma 2 (AIM2)	9477	5/c.712delA	NA	642/90	1	159062697	Frameshift deletion	NA
**Impact variants**												
R-HSA-844615	The AIM2 inflammasome	84	PYCARD	29108	2/c.C278G	NA	179/96	16	31202200	Nonsynonymous	1/10.47
R-HSA-75896	Plasmalogen biosynthesis	50	Glyceronephosphate O-acyltransferase (GNPAT)	8443	9/c.G1300A	11122266	364/158	1	231270961	Nonsynonymous	3/19.66
		66	Glyceronephosphate O-acyltransferase (GNPAT)	8443	9/c.C1240T	767514222	460/205	1	231270901	Nonsynonymous	3/14.27
		88	Alkylglycerone phosphate synthase (AGPS)	8540	c.A83T	764286061	135/97	2	177392872	Nonsynonymous	5/15.41
**STox group**												
**Functional variants**												
R-HSA-75205	Dissolution of fibrin clot	2	Serpin family F member 2 (SERPINF2)	5345	4/c.C169T	374446894	379/196	17	1745399	Stopgain	1/17.8
**Impact variants**												
R-HSA-77042	Formation of editosomes by ADAR proteins	94	Adenosine deaminase RNA specific (ADAR)	103	2/c.C577G	NA	467/261	1	154602065	Nonsynonymous	7/23.9

NA, not available.

QUAL parameter stands for a Phred-scaled score for the base assertion made for the variant allele; the AD parameter is the read depth, i.e., the number of reads mapped in this locus. Further information about the quality of the sequencing regarding the mutations of interest are available in **Supplementary Data 5**.

#### 3.2.2 STox Variant Enrichment Analysis

Regarding the STox variant enrichment analysis, two pathways, identified *in silico*, achived potential to translate the severe toxicity phenotype (marked in grey in **Tables 5**, **7** and listed in **Table 8**).

1) Dissolution of fibrin clot pathway (R-HSA-75205). A single stopgain variation on the serpin family F member 2 (SERPINF2) gene (rs374446894), was carried by patient #2, who devolveped a pulmonary thromboembolic event (**Table 5**).
2) Formation of editosomes by ADAR proteins (R-HSA-77042). A high-impact nonsynonymous variation (vi1.154602065, CAAD 23, impact score 7/10) on the adenosine deaminase RNA-specific (ADAR) gene, involved in Formation of editosomes by ADAR proteins, was carried by patient #94, who progressed to a high grade of fibrosis (**Table 7**). This variant also takes part in the mRNA editing: A to I conversion (R-HSA-75064) and C6 deamination of adenosine (R-HSA-75102) pathways; all together, these three pathways are related to mRNA editing.

Finally, we searched in our WES experimental dataset all the genetic variants, obtained trough GWAS, previously identified as associated to radiotherapy toxicity; no previous genetic variants associated with the studied phenotypes have been detected in the whole exome of NoTox and STox individuals (**Supplementary Data 4**).

## 4 Discussion

The employment of RHR in MPM has shown to confer a survival advantage but is associated with a nonnegligible toxicity profile in a fraction of patients (8, 11). This treatment should be offered to most mesothelioma-affected patients as it increases 2-year survival in inoperable patients to 58%, compared with the 28% rate reached by those who receive only CT or palliative radiotherapy (11). However, the toxicities associated with the treatment can have a relevant impact on the quality of life.

Pulmonary toxicity can heavily affect patient quality of life, causing mainly cough and dyspnea that last over time. In grades 1–2 dyspnea, there is a gradual worsening like exertional breathlessness, while grade 3 toxicity has an impact also on everyday life activities. Similarly, grades 1–2 pneumonitis is often paucisymptomatic affecting only mildly the quality of life, while grade 3 pneumonitis can have a permanent impact if it evolves in severe fibrosis (29), as observed in few cases in the present study.

The molecular events that lead to radiation-induced tissue toxicity/injury are complex and span a variety of biologic processes, including oxidative stress, apoptosis, inflammation, and release of proinflammatory and profibrogenic cytokines (30). The inflammatory environment induced by RT, worsened by pneumonitis and subsequent eventual fibrosis, could induce the development of fibrin clots, potentially leading to a thromboembolic event (31), a complication which occurred in 3 of our patients, as acute and late toxic effect.

The cause of radiation-induced normal tissue toxicity is multifactorial (13–15), and genetic factors have been hypothesized as playing a role in determining radiation response (32).

Despite the advances of genetic analyses based on next-generation sequencing technologies, although some GWAS and clinical sequencing of cancer patients have suggested that a number of variants in the DNA repair genes might underlie individual differences in chromosomal radiosensitivity within human populations (33), no translation to the clinical practice has been proposed so far. When considering the GWAS studies, the variants identified have not been replicated in different cohorts of patients, being the few genetic variants insufficient to fully describe a complex phenotype such as radiation response.

Our findings are not an exception, being the genetic variants identified through WES not specific of the two groups of patients analyzed (NoSTox and STox), consequently not allowing patients’ phenotype stratification. Moreover, when trying to find in our dataset the variants previously described in the GWAS already published, we did not observe any of them, once more reinforcing the fact that simple genetic variant analysis cannot explain multifactorial phenotypes.

So, being aware that a complex phenotype cannot be unravelled simply by comparing genetic variants distribution in two groups, also considering the low number of patients analyzed which contribute to statistical pressure and failure of association, we used a novel computational approach aimed at describing the molecular pathways specific for each group of patients. By doing so, thanks to the variable enrichment analysis, we have been able to identify pathways present just in the NoSTox or STox groups; a pathway signature has been recognized in NoSTox or STox.

Based on our variable enrichment analysis, here we describe the different pathways associated with the radiotherapy outcomes considered (none/tolerable or severe pulmonary toxicity), the relevance of which is also supported by literature.

### 4.1 None/Tolerable Toxicity Group

Four pathways, related to inflammatory and fibrotic processes, possibly involved in the development of lung toxicity, were *in silico* disrupted in the noSTox patients. Other disrupted pathways were related to more general biological processes not specifically related to the patients’ phenotypes. The four pathways are as follows:

1. TNFR1-induced NFkappaB signaling pathway (R-HSA-5357956)

Tumor necrosis factor alpha (TNF-α) is rapidly and persistently expressed in irradiated and adjacent tissue (34). It can trigger multiple signal pathways (35), including the signaling activity of NFkB, which has a strong proinflammatory action/function, and thus needs to be strictly controlled to prevent persistent inflammation. One mechanism that contributes to ensuring proper control of NFkB includes ubiquitination (36, 37). TNF-α has been implicated in radiation mucositis, enteritis, and dermatitis (30, 38–40), and its deficiency in a lung injury model has been shown to prevent symptoms of radiation pneumonitis (41).

A functional variation is found in TNFR1-induced NfkappaB signaling pathway: a frameshift insertion in the UBC gene. The protein encoded by this gene is a ubiquitinase (ubiquitin C). Although overall changes in ubiquitin cellular levels in response to ionizing radiation are still little known, Tang and colleagues showed that downregulation of ubiquitin C reduces radiation-induced expression of NF-kB and inhibits its translocation into the nucleus (42), suggesting that upon irradiation, de-ubiquitination silencing may suppress NF-kB-induced cellular response to radiation.

2. The AIM2 inflammasome pathway (R-HSA-844615)

A frameshift deletion in the AIM2 gene was detected in patient #16; also, a variant on PYCARD was observed in patient #84. AIM2 is an innate immune sensor that mediates assembly and activation of the inflammasome in response to double-stranded DNA breaks (43, 44). The DNA-sensing AIM2 inflammasome controls radiation-induced cell death and tissue injury (45). In a rat model of radiation pneumonitis (RP), radiotherapy increased the mRNA expression level of AIM2, which further triggered the release of IL-1β and induced RP (40), suggesting that the activation of the AIM2 inflammasome by radiotherapy may contribute to the pathogenesis of RP. Interestingly, AIM2 inflammasome deficiency has been shown to protect mice from radiation-induced small intestine syndrome as well as hematopoietic failure (45). Furthermore, the PYCARD (also known as ASC) gene encodes an adaptor protein that mediates assembly of large signaling complexes representing a critical step in initiating inflammasome responses (46).

3. The insulin receptor signaling cascade (R-HSA-74751) pathway was disrupted in 4 NoSTox patients

There is a high degree of structural homology between the insulin receptor (IR) and the insulin-like growth factor 1 (IGF-1) receptor (IGF-1R), which signals *via* many common mediators (47). Ionizing radiation activates several tyrosine kinase receptors involved in DNA damage response, including IGF-1R (48), possibly promoting a profibrotic state. Furthermore, dysregulation of the insulin-like growth factor (IGF) axis has been implicated in the pathogenesis of fibrosis in lung diseases (49–52). Insuline-like growth factor 2 (IGF-2) promotes fibrosis through IGF1R, IR, and IGF1R/IR, differentiates fibroblasts into myofibroblasts, decreases protease production and extracellular matrix degradation, and stimulates expression of two transforming growth factor β (TGF-β) isoforms, suggesting that IGF-2 may exert profibrotic effects *via* multiple mechanisms (53).

4. Plasmalogen biosynthesis (R-HSA-75896)

Recent evidence suggests that cholesterol-sphingolipid rafts might play a role in insulin signaling *via* IR (54). The potential contribution of lipids to insulin signaling is intriguing since the plasmalogen biosynthesis pathway has also been shown to have a potential impact on NoSTox patients’ phenotypes, carried by patients #50, #66, and #88 who did not develop severe pulmonary toxicities. Plasmalogens represent a class of phospholipids ubiquitously found in considerable amounts as constituents of mammalian cell membranes and significantly enriched in lipid rafts (55, 56), functional microdomains in cell membranes, which may affect signal transduction. Therefore, disrupting plasmalogen biosynthesis could affect trans-membrane signaling of insulin, thus making a functional bridge between this pathway and the pathogenetic mechanisms of radiation toxicity. Furthermore, Laiakis and colleagues (57) investigated the effects of ionizing radiation on the blood level of metabolites and lipids, showing that acute exposure to γ radiation in a mouse model induced specific mobilization of plasmalogens. Though the pathological role of plasmalogens and the biochemical pathways underlying their upregulation after radiation exposure remain to be elucidated, Braverman and Moser suggested a specific serum lipidomic biosignature as an indicator of radiation exposure (58).

**Figure 1:**
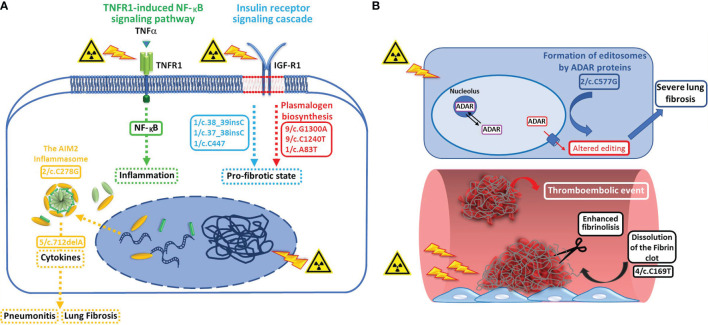
**(A)** Variant enrichment analysis performed on individuals who developed none/tolerable radiation-induced pulmonary toxicity suggests that functional impairment (dotted arrows) in pathways involved in the control of the radiation-induced expression of NF-kB (green dotted arrow = TNFR1-induced NFkB signaling pathway, functional variant related: 2/c.2051dupG) and AIM2 inflammasome activation (yellow dotted arrow = the AIM2 inflammasome, functional variant related: c.712delA, impact variant related: c.C278G), and of the insulin-like growth factor (IGF) axis (blue dotted arrow = insulin receptor signaling cascade, functional variants related: 1/c.38_39insC, 1/c.37_38insC, 1/c.37_38insC, and 1/c.C447), possibly correlated to an impaired plasmalogen biosynthesis (red dotted attow = plasmalogen biosynthesis, impact variants related: 9/c.G1300A, 9/c.C1240T, and 1/c.A83T), could play a role in preventing the development of acute radiation pneumonitis and chronic radiation-induced pulmonary fibrosis. **(B)** Variant enrichment analysis performed on individulas who developed severe radiation induced pulmonary toxicity suggests that, by affecting both the fibrinolytic activity (white arrow = dissolution of fibrin clot, functional variant involved: 4/c.C169T) and RNA editing pathways (light blue arrow = formation of editosomes by ADAR proteins, impact variant involved: 2/c.C577G), specific variants in genes involved in these pathways could be responsible of the severe toxicity events reported by some patients after irradiation.

Based on the pathways findings in NoSTox subjects, we hypothesized that functional impairment in the control of the radiation-induced expression of NF-kB, mainly in/of the ubiquitination/deubiquitination mechanisms, together with an impairment of the AIM2 inflammasome pathway and a dysregulation of the insulin-like growth factor (IGF) axis (possibly correlated to a impaired plasmalogen biosynthesis), could modulate radiation-induced lung inflammation preventing development of acute radiation pneumonitis and chronic radiation-induced pulmonary fibrosis (**Figure 1**).

### 4.2 Severe Toxicity Group

The same approach based on the detection-disrupted pathways was used for the Stox group.

Two disrupted pathways, with potential impact on Stox patients’ phenotypes were found:

1. The dissolution of the fibrin clot pathway (R-HSA-75205)

One variant related to this this pathway was identified: a stopgain variant in the SERPINF2 gene in patient #2, who developed a thromboembolic event.

Irradiation activates procoagulant activity and reduces fibrinolytic activity (59) in animal models of thoracic irradiation, suggesting the involvement of coagulation and fibrinolysis in radiation-induced pulmonary injury (60). The SERPINF2 gene encodes a member of the serpin family of serine protease inhibitors, the alpha 2 antiplasmin (α2-AP), which, together with the plasminogen activator inhibitor-1 (PAI-1), is the principal direct inhibitor of fibrinolytic activity. Alpha2-antiplasmin plays a significant role in acute pulmonary embolism (61) and patients with pulmonary embolism have been shown to display higher rate of fibrin clot degradation (62). Impairment of Alpha2-antiplasmin activity by the stopgain variant in the SERPINF2 gene, by possibly enhancing fibrinolytic activity, could then be involved in the development of the pulmonary thromboembolic event observed in patient 2#.

2. Formation of editosomes by ADAR proteins (R-HSA-77042)

A high impact nonsynonymous variant in the ADAR gene, involved in the formation of editosomes by ADAR proteins, was carried by patient #94, who progressed to high-grade fibrosis.

The posttranscriptional modification of RNA is a key process controling the output of the genome, and the deamination of adenosines (A) to inosines (I) is the prominent RNA editing event in humans, catalyzed by the adenosine deaminase acting on RNA (ADAR) family of proteins (63). The effects of radiation on RNA editing are poorly understood; Liu and colleagues reported that upon/after α-particle radiation, RNA editing sites change greatly and their total amount decreases after radiation (64). It has been determined that the expression of ADAR is tissue specific, the lung being the second most highly expressing site, in terms of tissue expression (65, 66). The RNA editome (R-HSA-77042) analysis of idiopathic pulmonary fibrosis (IPF) and normal lungs revealed increased editing frequency in IPF compared with normal lungs, suggesting a role for dysregulated editing in IPF pathogenesis (67). It has been found that the expression of ADAR1 and ADAR2 is downregulated in fibroblasts from patients with IPF (68). The changes in expression levels of ADAR1 and ADAR2 may represent an important controling mechanism in IPF, regulating the processing of key miRNAs such as miRNA-21. Overexpression of miRNA-21 in lung tissue and pulmonary fibroblasts from patients with IPF may be due to defective editing by ADAR (66). This miRNA targets antifibrosing protein synthesis such as SMAD family member 7, TGF beta receptor 2 (TGF-βR2), TIMP metallopeptidase inhibitor 3 (TIMP3), and vascular endothelial growth factor A (VEGF-A) (69, 70). Crosstalk among impact pathways which control RNA editing (R-HSA-75064 and R-HSA-77042) could contribute to the induction of the severe lung fibrosis observed in patient #94.

Taken together the pathway findings of Stox patients, lead us to hypothesize that specific variants in genes affecting both the fibrinolytic activity and RNA editing pathways, could be responsible of the severe toxicity events reported, after irradiation, by some patients of the Stox group, who present these specific variants (**Figure 1**).

## 5 Conclusion and Future Perspectives

Our WES findings did not show any specific genetic variant associated with NoSTox and Stox phenotypes, thus confirming that the association study approach is not useful when considering complex phenotype such as the response to radiotherapy; however, the results obtained by variant enrichment analysis indicate different pathways signatures characterizing NoSTox and Stox patients, allowing to formulate hypotheses on the protection from side effects derived from radiotherapy as well as factors predisposing to a worst response to the treatment. Of course, we are aware that our study suffers of the limitation related to the small number of patients analyzed, and the lack of trascriptomic data, not available due to the impossibility to collect lung/pleural biological samples for RNA sequencing, to double check our hypothesis.

The possibility to identify patients that, based on their signatures of pathways, could better respond to radiotherapy represents a future translational application, once the signatures validated on other groups of patients. By allowing early identification of patients at risk for treatment-dependent pulmonary toxicity, the pathway-based predictive tool could play a role in the design of new therapeutic combinations, including immunotherapy and RT. Indeed, first-line nivolumab plus ipilimumab treatment has been recently proposed in unresectable MPM, to improve OS compared with chemotherapy. The potential synergy of RT and immunotherapy, already observed in other cancers (71), could not only further improve the clinical response in MPM but also increase the risk of side effects, shared by both treatments, such as pneumonitis (72). In this context, the pretreatment genomic characterization could help to prevent the development of severe side effects by contributing to the definition of a personalized treatment.

## Data Availability Statement

The datasets presented in this study can be found online at SRA repository by PRJNA768626 id: https://www.ncbi.nlm.nih.gov/sra/PRJNA768626.

## Ethics Statement

This prospective study, involving human participants, was reviewed and approved by the local Ethical Committee (Comitato Etico Indipendente del CRO di Aviano, CRO-2013-38). The patients provided their written informed consent to participate in this study. The CRO biobanking service managed and stored all biological samples before use for the present project (authorization for analyses obtained through protocol number 6825/D).

## Author Contributions

SC and VB conceived the original idea and drafted the manuscript. AR acquired the data and contributed to the interpretation of the results. RM and LB performed genetic analysis and interpretation of the data. ElM and GZ contributed to the final version of the manuscript, provided critical feedback, and helped shape the research. AS, MT, EmM, and PZ helped supervise the project. All authors read and approved the final version.

## Funding

This work was supported by grants from the Italian League for the Fight Against Cancer (LILT), ASSOCIAZIONE ISONTINA LILT (Bando di Ricerca sanitaria 2017-programma 5 per mille anno 2015) and from Municipality of Monfalcone (Gorizia).

## Conflict of Interest

The authors declare that the research was conducted in the absence of any commercial or financial relationships that could be construed as a potential conflict of interest.

## Publisher’s Note

All claims expressed in this article are solely those of the authors and do not necessarily represent those of their affiliated organizations, or those of the publisher, the editors and the reviewers. Any product that may be evaluated in this article, or claim that may be made by its manufacturer, is not guaranteed or endorsed by the publisher.
