# Supplementary material for: Biological Pathways Associated With the Development of Pulmonary Toxicities in Mesothelioma Patients Treated With Radical Hemithoracic Radiation Therapy: A Preliminary Study
Source: Front Oncol. 2021 Dec 22;11:784081. doi: 10.3389/fonc.2021.784081 (PMC8728021; doi:10.3389/fonc.2021.784081)
Supplement: Supplementary file 1 [file DataSheet_1.docx]

**Supplementary data**

****For large tables please see files enclosed:***

***784081_Borelli_Table 1.XLSX*** *for Supplementary TableS2a*

***784081_Borelli_Table 2.XLSX*** *for Supplementary Table S2b*

***784081_Borelli_Table 3.XLSX*** *for Supplementary Table S2c*

***784081_Borelli_Table4.XLSX*** *for* *Supplementary Table S2d.*

***784081_Borelli_Table 5.XLSX*** *for* *Supplementary Table S3a.*

***784081_Borelli_Table 6.XLSX*** *for Supplementary Table S3c.*

***784081_Borelli_Table 7.XLSX*** *for* *Supplementary Tables S5 a and b.*

**Supplementary data 1: descriptive information about exomic analysis of the 49 patients with mesothelioma.**

#### Supplementary 1a: distribution of variants from Exome of STox and NoSTox groups

The average number of variants per sample in the NoSTox group was 173,812 with 35,097 (21.34% of the total) being specific exon variants (ExonVars). For the STox group, the average number of mutations per sample was 176,339 with 35,132 (19.92%) ExonVars. Subsequently, ExonVars were categorized in two groups: the first group included the so-called Functional Variants (FunctVar), comprising frameshift insertion/deletion, start loss and stop gain/loss variants. The second group comprised the Impact Variants (ImpactVar), which are the variants predicted as ‘damaging’ by at least one of the following softwares: SIFT, Polyphen2, LRT, Mutationtaster, Mutationassessor, FATHMM, Provean, Metasvm, Metalr and M_cap or CADD equals or higher than 15 (Table S1a). For the NoSTox group, on average, we found 326 FunctVar and 4,024 ImpactVar, whereas, for the STox group, we identified 328 and 3,996 FunctVar and ImpactVar, respectively (Table S1a).

| **Table S1a: distribution of variants from Exome of STox (Severe toxicity) and NoSTox (no or tolerable toxicity) groups of patients.** | | | | | |
| --- | --- | --- | --- | --- | --- |
|  | **All** | **ExonVar** | **FuncVar** | **ImpactVar** | **Rare** |
| NoTox | 173812 | 35097 | 326 | 4024 | 5682 |
| STox | 176339 | 35132 | 328 | 3996 | 5740 |

***FunctVar: Functional variant; ImpactVar: Variants with a prediction impact higher than 1 or CADD >=15.*** *Rare: variant with minor allele frequency (MAF)lower than 0.05.*

#### Supplementary 1b and 1c: distribution (%) of variant location and function from Exome Report of both groups (STox and NoSTox)

As expected, the majority of variants were located on Exonic region, followed by UTR3, UTR5 and ncRNA in both STox (Severe toxicity) NoSTox (none or tolerable toxicity) groups of patients (Figure S1a).

***
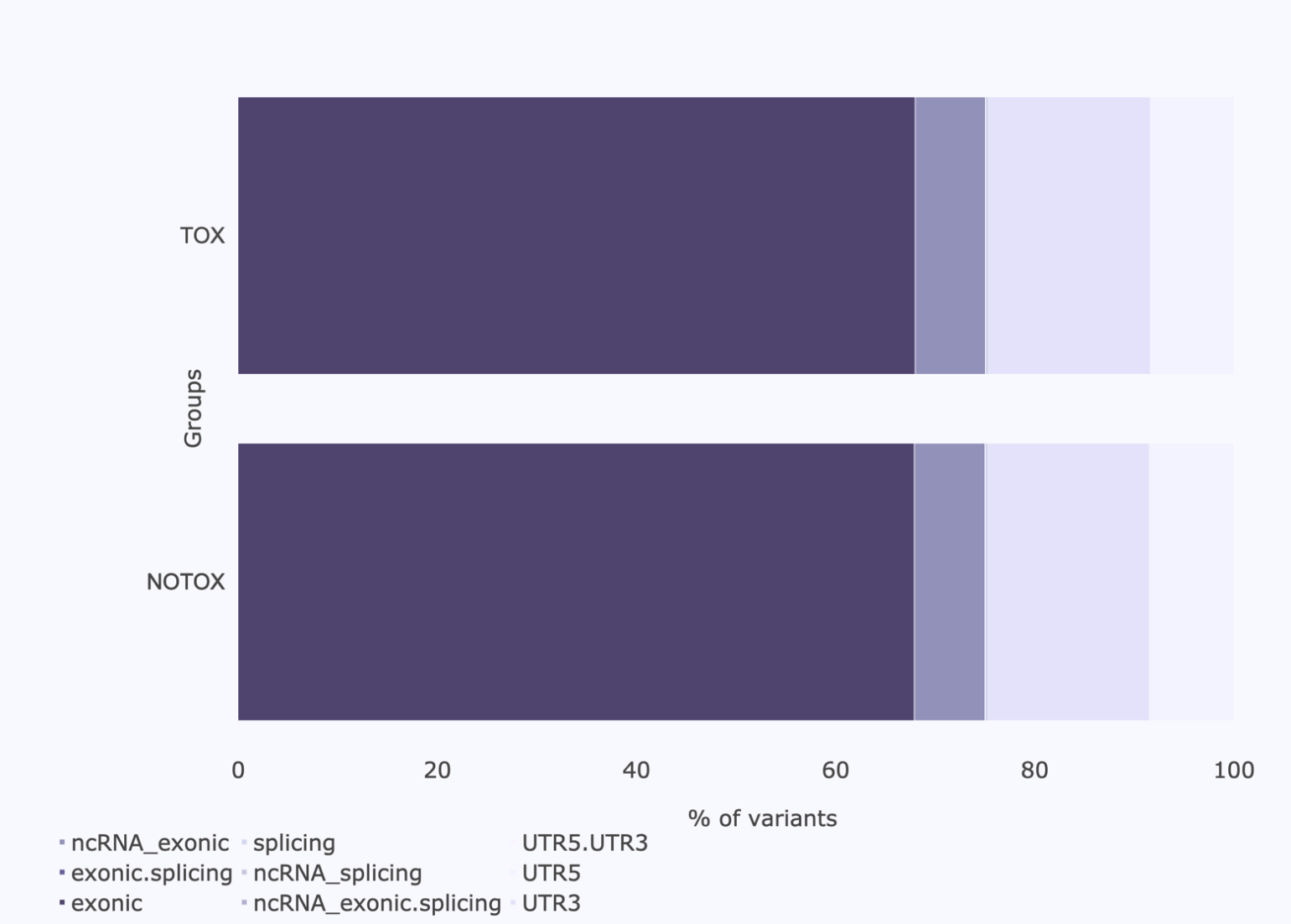
***

**Figure S1a: distribution of variant location (%) from Exome Report of both groups. STox (Severe toxicity) and NoSTox (none or tolerable toxicity).**

Exploiting Exonic Variants (ExonVars) functionality, around 46% were annotated as Nonsynonymous variant (nsVar) and 49% were synonymous (sVar) (Figure S1b).

**
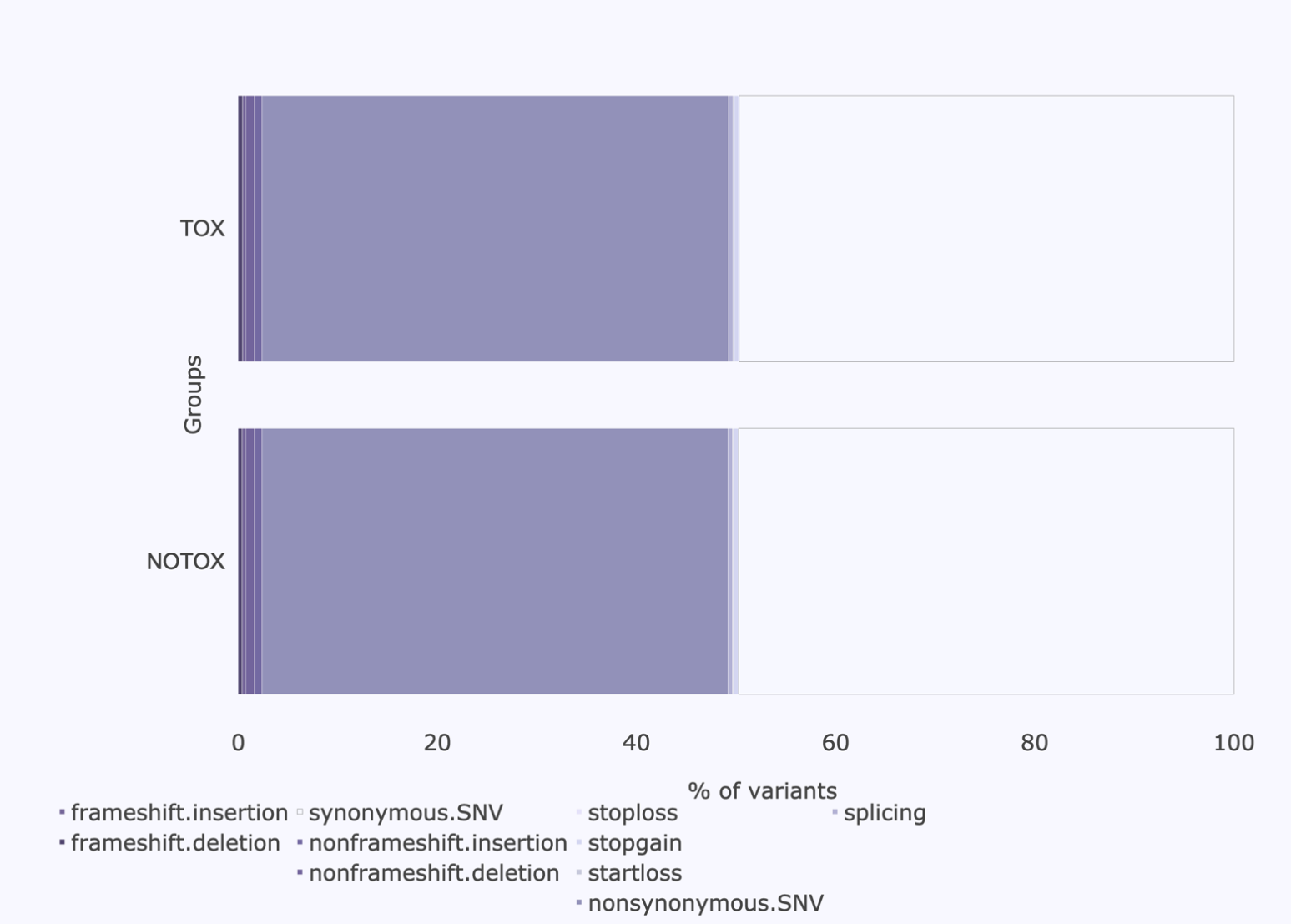
**

**Figure S1b: distribution of variant function on groups STox (Severe toxicity) and NoSTox (none or tolerable toxicity*).***

#### Supplementary 1d: distribution of genes carrying variants of STox and NoSTox groups.

Then, we considered the genes in which variants were present; in the NoSTox group, the average number of genes bearing variants was 17,983 with 12468 (69.72%) presenting at least one ExonVar; in the STox group variants were detected in 18,021 genes, with 12,466 (69.17%) genes carrying at least one ExonVar. Two-hundred sixty-five genes had at least one Functional variant, in the NoSTox group, and 266 in the STox group. The number of genes with at least one Impact variant was 2,803 and 2,796 for the NoSTox and STox groups, respectively. The number of exclusive genes carrying functional or impact variants in the STox group were 17 and 188, respectively.

| **Table S1b: distribution of genes carrying variants of STox (Severe toxicity) and NoSTox (none or tolerable toxicity) groups of patients.** | | | | | |
| --- | --- | --- | --- | --- | --- |
|  | **All** | **ExonVar** | **FuncVar** | **ImpactVar** | **Rare** |
| NoTox | 17983 | 12468 | 265 (9)* | 2803 (51)* | 3363 |
| STox | 18021 | 12466 | 266 (17)* | 2796 (188)* | 3378 |

In bractless are the quantity of exclusive genes carrying mutations. Rare: variant with minor allele frequency (MAF)lower than 0.05.

**Supplementary data 2: comparative description about common variants and genes of 49 patients with mesothelioma.**

**Supplementary 2a and 2b:**

We then filtered NoSTox and STox common variants to retrieve only the common variants exclusive to the STox group. We found 18 FunctVar (Table 2a) and 211 ImpactVar (Table 2b).

| ***Table S2a: distribution of functional variants (FunctVar) that are common and exclusive in all STox (Severe toxicity) and not in NoSTox (none or tolerable toxicity) group of mesothelioma patients.** | | | |
| --- | --- | --- | --- |
| datatables | | | |
| **rsID** | **Genotype** | **NoTox** | **STox** |
| **…** | **…** | **…** | **…** |
| ***File: 784081_Borelli_Table 1.XLSX*** | | | |

| ***Table S2b: distribution of common impact variants (ImpactVar) that are common and exclusive in all in STox (Severe toxicity) and not in NoSTox (none or tolerable toxicity) group of mesothelioma patients.** | | | |
| --- | --- | --- | --- |
| datatables | | | |
| **rsID** | **Genotype** | **NoTox** | **STox** |
| **…** | **…** | **…** | **…** |
| ***File: 784081_Borelli_Table 2.XLSX*** | | | |

#### Supplementary 2c and 2d:

We then filtered NoSTox and STox common variants to retrieve only the common variants exclusive to the NoSTox group. We found 88 FunctVar (Table 2c) and 51 ImpactVar (Table 2d).

| ***Table S2c: distribution of common functional variants (FunctVar) presented in NoSTox (none or tolerable toxicity) group of mesothelioma.** | | | |
| --- | --- | --- | --- |
| datatables | | | |
| **rsID** | **Genotype** | **NoTox** | **STox** |
| **…** | **…** | **…** | **…** |
| ***File: 784081_Borelli_Table 3.XLSX*** | | | |

| ***Table S2d: distribution of common impact variants (ImpactVar) presented in NoSTox (none or tolerable toxicity) group of mesothelioma.** | | | | |
| --- | --- | --- | --- | --- |
| datatables | | | | |
| **rsID** | **Genotype** | **NoTox** | **STox** | |
| **…** | **…** | **…** | **…** |  |
| ***File: 784081_Borelli_Table 4.XLSX*** | | | | |

**Supplementary data 3: pathway descriptive analysis from 49 patients with mesothelioma classified by radiotoxicity response.**

**Supplementary 3a**

| ***Table S3a: list of the variants present in the functional pathways found in the NoSTox (no or tolerable toxicity) group.** | | | | | |
| --- | --- | --- | --- | --- | --- |
| **DB_ID** | **rsID** | **Gene Symbol** | **ENTREZID** | **Exonic Function** | **Prediction** |
| **…** | **…** | **…** | **…** | **…** | **…** |
| ***File: 784081_Borelli_Table 5.XLSX*** | | | | | |

DB_ID: Reactome ID.

**Supplementary 3b:**

| **Table S3b: list of the variants present in the functional pathways found in the STox (Severe toxicity) group.** | | | | | |
| --- | --- | --- | --- | --- | --- |
| **DB_ID** | **rsID** | **Gene Symbol** | **ENTREZID** | **Exonic Function** | **Prediction** |
| R-HSA-75205 | rs374446894 | SERPINF2 | 5345 | stopgain | 1 |
| R-HSA-72649 | vi15.44537196 | EIF3J | 8669 | nonframeshift insertion | 0 |
| R-HSA-72649 | vi8.100709705 | PABPC1 | 26986 | frameshift deletion | 0 |
| R-HSA-72649 | vi19.38626091 | EIF3K | 27335 | stopgain | 2 |
| R-HSA-72695 | vi15.44537196 | EIF3J | 8669 | nonframeshift insertion | 0 |
| R-HSA-72695 | vi19.38626091 | EIF3K | 27335 | stopgain | 2 |
| R-HSA-72702 | vi14.103338419 | EIF5 | 1983 | nonframeshift insertion | 0 |
| R-HSA-72702 | vi15.44537196 | EIF3J | 8669 | nonframeshift insertion | 0 |
| R-HSA-72702 | vi19.38626091 | EIF3K | 27335 | stopgain | 2 |

#### Supplementary 3c:

| *Table S3c: list of the variants present in the impact pathways found in the NoSTox (no or tolerable toxicity) group. | | | | | |
| --- | --- | --- | --- | --- | --- |
| datatables | | | | | |
| **DB_ID** | **rsID** | **Gene Symbol** | **ENTREZID** | **Exonic Function** | **Prediction** |
| **…** | … | … | … | … | … |
| \| ***File: 784081_Borelli_Table 6.XLSX*** \| \| --- \| | | | | | |

**Supplementary 3d:**

| **Table S3d: list of the variants present in the impact pathways found in the STox (Severe toxicity) group. *File: Table_S3d.xlsx*** | | | | | |
| --- | --- | --- | --- | --- | --- |
| datatables | | | | | |
| **DB_ID** | **rsID** | **Gene Symbol** | **ENTREZID** | **Exonic Function** | **Prediction** |
| R-HSA-75064 | vi1.154602065 | ADAR | 103 | nonsynonymous SNV | 7 |
| R-HSA-75102 | vi1.154602065 | ADAR | 103 | nonsynonymous SNV | 7 |
| R-HSA-77042 | vi1.154602065 | ADAR | 103 | nonsynonymous SNV | 7 |


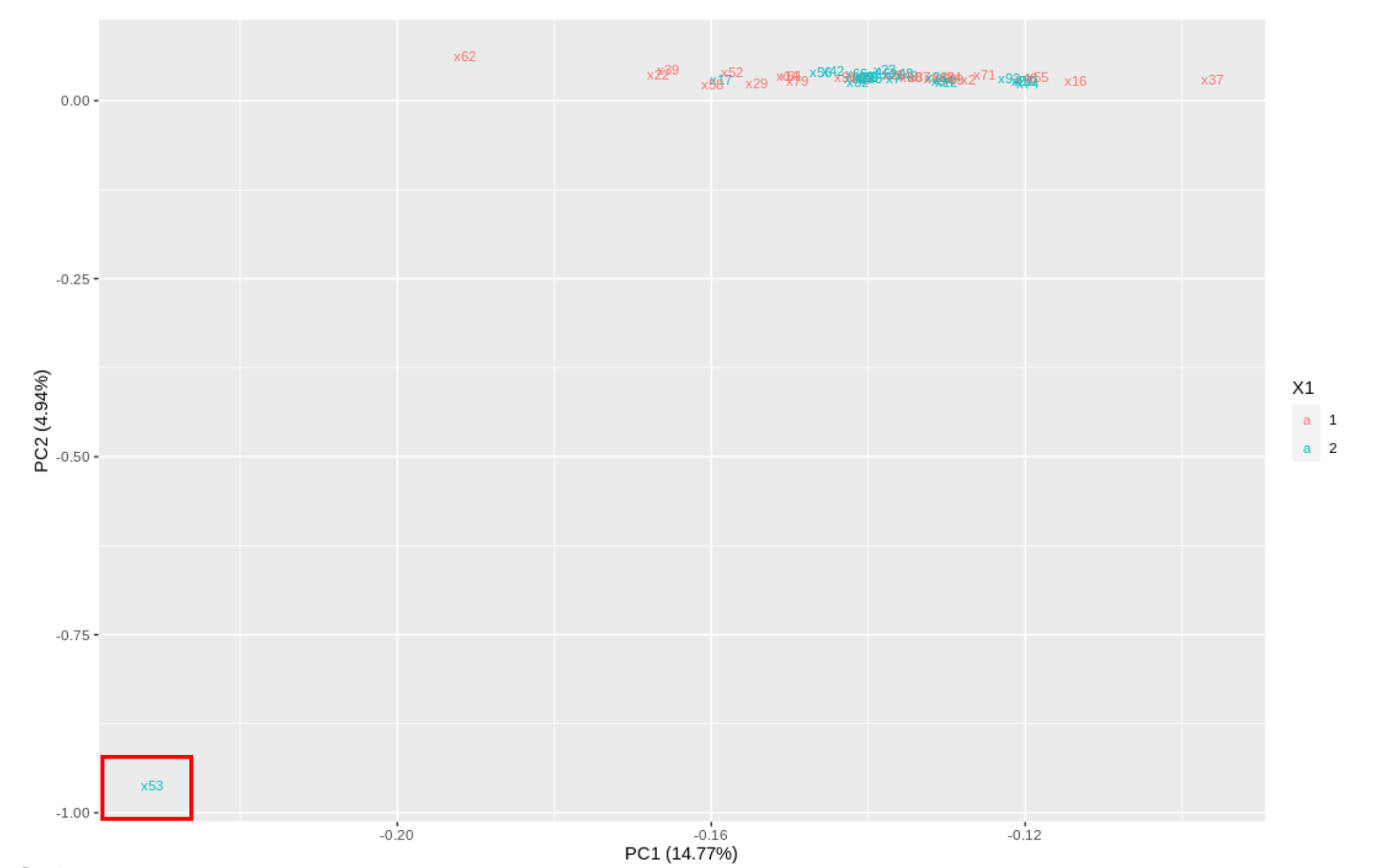


**Figure S3. Principal component analysis (PCA) of genetic variants distribution in the NoSTox (red x) and STox (blue x) groups. The variant distribution is homogeneous, no differences between the NoSTox and STox groups can be observed. Patient 53 (red square) from the STox group is quite distant from all other patients, regardless of whether they were NoSTox or STox, thus suggesting the presence of a specific genetic signature for this individual that is completely independent of the studied phenotype.**

# Supplementary data 4: comparative description with other genomic studies about radiotoxity in cancer treatment

#### Supplementary 4a and 4b:

We selected 163 variants previously associated with radiotoxicity response during cancer treatment. The firsts groups of variant were from gene-based association studies (n=5). The second one represents variants reported by the Radiogenomic Consorcium (n=51). And the last one was from the GWAS catolog response to radiantion trait (GO_0009314) (n=107). The analysis was based on two approaches: (1) common genes shared by each group and (2) all variants from each group was included. None of the 163 variants were found in STox or NoTox group (Figure S4a). By the second approach, the variant rs9484606 of GO_0009314 trait was present on NoTox group and three variants (rs17599026, rs4742269, rs76273496) of GO_0009314 trait was present on STox patients (Figure S4b).


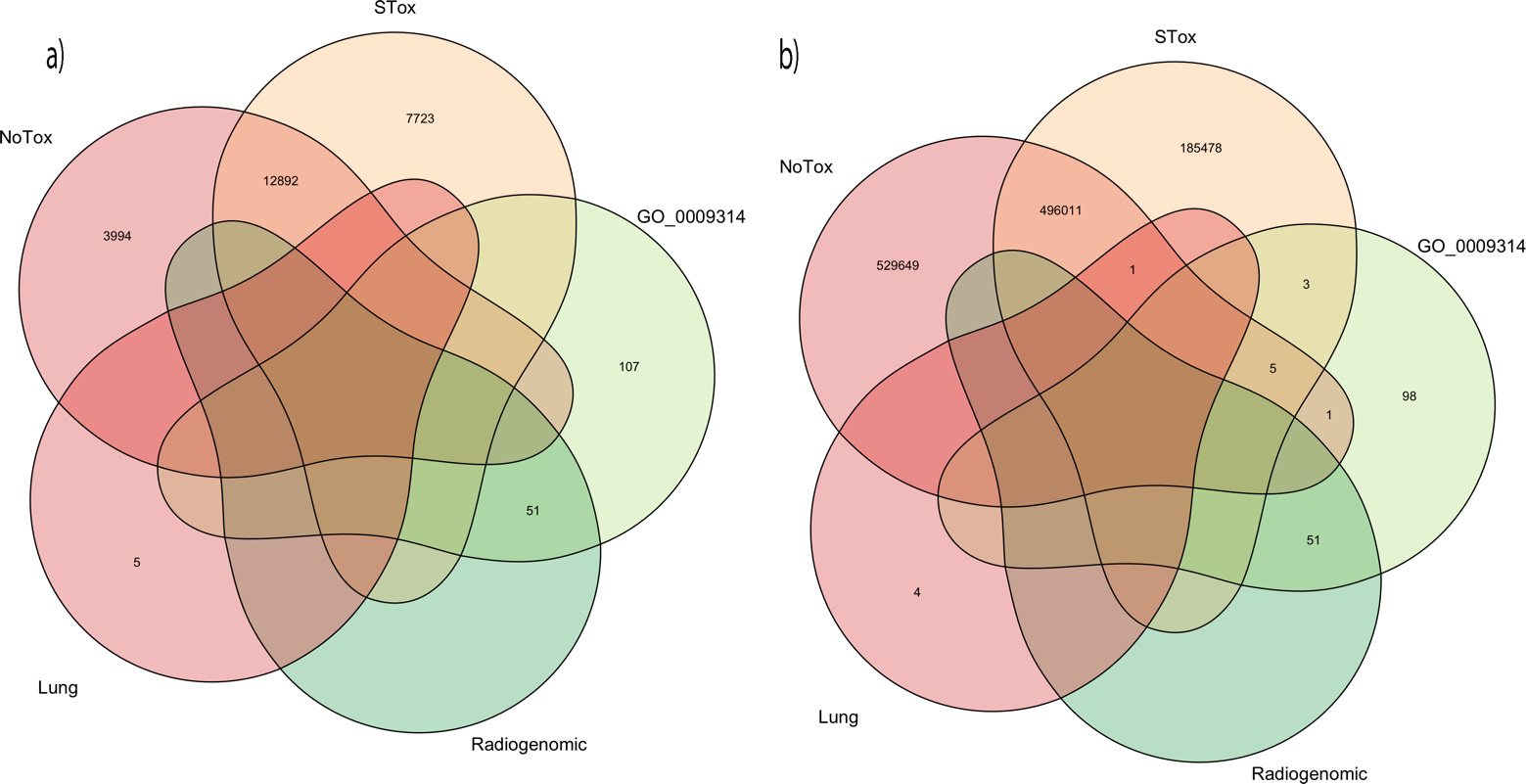


**Figure S4**

**a: Venn diagram of common variants for STox (Severe toxicity), NoSTox (none or tolerable toxicity) and selected variants related to radiotoxicity.**

**b:Venn diagram of all variants for STox (Severe toxicity), NoSTox (none or tolerable toxicity) and selected variants related to radiotoxicity.**

#### Supplementary 4c

Then we selected the variants derived from Venn diagram analysis to count how many patients of each group carry the respective variant (Table S4a and S4b).

| **Table S4a: distribution of variants previously related to radiotoxicity response in NoSTox (none or tolerable toxicity).** | | |
| --- | --- | --- |
| **Variant** | **NoTox** | **STox** |
| rs9484606 | 1 | 0 |
| rs9484606 | 32 | 16 |

| **Table S4b: Distribution of variants previously related to radiotoxicity response in STox (Severe toxicity).** | | |
| --- | --- | --- |
| **Variant** | **NoTox** | **STox** |
| rs17599026 | 33 | 13 |
| rs17599026 | 0 | 2 |
| rs17599026 | 0 | 1 |
| rs4742269 | 0 | 2 |
| rs4742269 | 33 | 14 |
| rs76273496 | 0 | 1 |
| rs76273496 | 33 | 15 |

**Supplementary data 5: Quality of Exome Sequencing.**

| **Table S5a: the QUAL parameter is referred to the Phred-scaled score for the base assertion made for the variant allele. The QUAL cut off was equal or higher than 30.** | | | | | | | |
| --- | --- | --- | --- | --- | --- | --- | --- |
| Patient.id | Min. | 1st Qu. | Median | Mean | 3rd Qu. | Max. | SD |
| … | … | … | … | … | … | … | … |
| ***File: 784081_Borelli_Table 7.XLSX***  ***(sheet 1)*** | | | | | | | |

| **Table S5b: the AD parameter is the read depth, i.e. the number of reads mapped in this locus. The AD cut off was equal or higher than 10.** | | | | | | | |
| --- | --- | --- | --- | --- | --- | --- | --- |
| Patient.id | Min. | 1st Qu. | Median | Mean | 3rd Qu. | Max. | SD |
| … | … | … | … | … | … | … | … |
| ***File: 784081_Borelli_Table 7.XLSX***  ***(sheet 2)*** | | | | | | | |
